# Global cerebral edema and brain metabolism after subarachnoid hemorrhage

**DOI:** 10.1186/cc9747

**Published:** 2011-03-11

**Authors:** R Helbok, J Claassen

**Affiliations:** 1 Medical University, Innsbruck, Austria; 2 Columbia University Medical Center, New York, USA

## Introduction

Global cerebral edema (GCE) is common amongst poor-grade subarachnoid hemorrhage (SAH) patients and associated with poor outcome. Currently no targeted therapy exists largely due to an incomplete understanding of the underlying mechanisms.

## Methods

This is a prospective observational study including 39 consecutive poor-grade SAH patients with multimodal neuromonitoring. Levels of microdialysate lactate/pyruvate ratio (LPR), episodes of cerebral metabolic crisis (MC; LPR >40 and brain glucose <0.7 mmol/l), brain tissue oxygen tension (PbtO_2_), cerebral perfusion pressure (CPP), and transcranial Doppler sonography flow velocities were analyzed.

## Results

Median age was 54 years (45 to 61) and 62% were female. Patients with GCE on admission (*n *= 24, 62%) had a higher incidence of MC in the first 12 hours of monitoring than those without GCE (*n *= 15; 15% vs. 2%, *P *< 0.05) and during the total time of neuromonitoring (20% vs. 3%, *P *< 0.001). There was no difference in PbtO_2 _and CPP between the groups; however, in patients with GCE a higher CPP was associated with lower LPR (*P *< 0.05). Episodes of crisis were associated with poor outcome (modified Rankin Score 5 or 6, *P *< 0.05).

## Conclusions

In poor-grade SAH patients, GCE is associated with early brain metabolic distress. Optimizing cerebral blood flow and homeostasis early after SAH may prove beneficial for patients with GCE.
